# Medical Items

**Published:** 1892-07

**Authors:** 


### *MEDICAL ITEMS.*

The New York Post-Graduate Medical School and Hospital will erect new buildings at the corner of Second avenue and 20th street.

The Pasteur Institute in New York City, established by Dr. Paul Gibier for the treatment of hydrophobic patients, has been closed for want of funds.

Dr. Henry T. Formad, of Philadelphia, the distinguished expert in microscopy and toxicology, died June 10th, at the age of forty-five. He was a Russian by birth and was exiled on account of Nihilistic tendencies.

In opposition to the statement first made by Dr. Weir Mitchell that chorea does not occur in the negro race, Dr. Aiken, of Laurens, S. C., reports a case (*Medical News*, May 14th). Patient was negro boy (not mulatto) between twelve and fourteen years old. He was given the usual treatment of Fowler's solution, and improved.

Prof. T. G. Richardson, of Tulane University, New Orleans, died May 26. He had been actively connected with the medical department of the University for thirty-one years; fourteen years as professor of anatomy, seventeen years as professor of surgery, and for twenty of these years he was dean. On May 30th, his colleagues of the medical faculty paid a high tribute to the memory of Prof. Richardson in a series of resolutions which he wholly deserved. At the next annual commencement, April 5, 1893, memorial addresses upon his life and services will be delivered.

MR. ROBERT A. BARNES, of St. Louis, recently deceased, bequeathed about $900,000 for the erection and maintenance of a hospital in that city. It will be conducted under the auspices of the Southern Methodist church, but will dispense its charity regardless of creed or nationality.

---

IT gives us pleasure to speak a word of encouragement in behalf of our young contemporary, the *Medical Fortnightly*, of St. Louis. But six month old, as yet, it is hale and hearty and apparently well nourished. It has come to our desk every two weeks, and we rarely fail to read it. Our best wishes to the *Fortnightly*, and our congratulations to its live and "hustling" editor!

---

WE take pleasure in offering the *amende honorable* for an unjust reflection on the standing of Dr. Emory Lanphear, of Kansas City, Mo., which appeared in our last issue. We were incautiously led into an error which we much regret by an item found in the columns of an unhealthy exchange from the Southwest. Our quotation was unfortunate and ill-advised, and we hope Dr. Lanphear will pardon our indiscretion which, we are sure, did him much injustice.

---

THE Hon. Jacob H. Gallinger, United States Senator from New Hampshire, is advocating a means in the Senate to establish a national sanitarium for the treatment of pulmonary diseases. The bill proposes to erect a sanitarium on public lands in one of the western territories, and in view of the fact that the death rate from pulmonary diseases in New Mexico is only 3 per cent., as compared with 25 per cent. in New England, and 20 per cent. in the Middle States, the Senator thinks that the former would be the most favorable location.

A DEAD BEAT.—In the publication of this journal we have carefully avoided taking any advertisements except from first-class, reliable, meritorious houses, and for the time we have been publishing the journal we have to report but one first-class fraud. The Barr Electric Manufacturing Co., of 17 and 19 Broadway, N. Y., is not a case of "*honest inability to pay*," if we are correctly informed, but it is a concern determined to beat medical journals out of their legitimate dues. We therefore warn the readers of the "*Age*" and the medical journals of the country, that in our opinion this Barr Electric Manufacturing Co., of 17 and 19 Broadway, N. Y., is a rotten, thieving, malicious, designing, scheming fraud of the first water, and we regret that the files of our journal show that we run their advertisement for three months.—*The Alabama Medical and Surgical Age.*

---

THE alleged murder of his wife by medical student Carlyle W. Harris, of New York, and his trial and conviction, will furnish one of the *causes celebrés* to medical jurisprudence. In his cell in the Tombs the condemned has recently written the following very pretty lines:

SLEEP.

Thou tender, loving mistress of mankind,
Soother of pain by touch of soft caress,
Come near and press my eyelids with thy lips,
And lull me into sweet forgetfulness.

Smooth from my brow the furrowed line of care,
Upon my forehead let thy cool palm lie,
My aching forehead to the throbbing brain,
Sing thou a sweet lullaby.

Sing me a dream of groves and leafy trees,
With twinkling rivulet and flow'ry bush,
And bright-hued daisies nodding to the breeze,
Beneath the flight of bobolink and thrush.

Waft to mine ears the murmur of the brook,
Along whose banks the trailing willows weep,
Then with thy soft, warm arms encircling me,
Pillow my head upon thy breast, O Sleep.

